# Age-specific Epstein-Barr virus seroprevalence in Costa Rica

**DOI:** 10.1371/journal.pone.0362125

**Published:** 2026-11-10

**Authors:** Sabrina Acosta-Egea, Elexandra Barboza Arguedas, Eva Villalobos Chacón, Romain Fantin, Carolina Porras, Viviana Loria, Juan Carlos Venegas, Connie M. Weston, Sarah S. Jackson, Arturo Abdelnour, Amada Aparicio, Alejandro Calderón, Daniela Mora, Rebeca Ocampo, Roy Wong-McClure, Melvin Morera, Roberto Castro, Henry H. Balfour, Jr, Rolando Herrero, Allan Hildesheim

**Affiliations:** 1 Agencia Costarricense de Investigación Biomédica, Fundación INCIENSA (ACIB-FUNIN), San José, Costa Rica; 2 Hospital Nacional de Niños “Dr. Carlos Sáenz Herrera” (HNN), San José, Costa Rica; 3 Department of Laboratory Medicine and Pathology, Department of Pediatrics, University of Minnesota Medical School, Minneapolis, Minnesota, United States of America; 4 National Cancer Institute (NCI), Bethesda, Maryland, United States of America; 5 Caja Costarricense de Seguro Social (CCSS), San José, Costa Rica; 6 Ministerio de Salud, San José, Costa Rica; Fondazione Don Carlo Gnocchi, ITALY

## Abstract

Epstein-Barr Virus (EBV) is a common herpesvirus linked to cancers and autoimmune diseases. The age, socioeconomic status and geographic distribution of EBV infection are varied. This study evaluated age-specific EBV antibody prevalence in Costa Rica among 2,990 participants in a population-based cohort that enrolled in 2020/1. Seropositivity prevalence was age-dependent, with rates over 80% in adolescents, and higher than 90% in adults 20 + . Geometric mean levels (GML) of antibodies among seropositives also rose with age, peaking in older adults. The age at seroconversion was slightly younger in boys than in girls and for individuals in rural/mixed areas, but seropositivity of 90% or more was found in both sexes and all regions of residence in adults. Among seropositives, there was a tendency for women to have higher GML compared with men. Our findings provide information regarding the age-specific distribution of EBV in Costa Rica, which is useful for planning future health measures to reduce the burden of EBV-associated conditions.

## Introduction

Epstein-Barr Virus (EBV) is a ubiquitous herpesvirus that establishes a lifelong latent infection within B-cells after primary infection [1]. Normally, infection with EBV occurs early in life and often has no symptoms [2]. EBV is associated with the development of infectious mononucleosis (IM) as well as a number of lymphoproliferative diseases and malignancies [3]. EBV has also in more recent years been associated with multiple sclerosis (MS) [4]. The age of primary infection influences the clinical manifestations that develop [2].

While EBV is common, its infection patterns vary by socioeconomic status (SES) factors within and across geographic regions. EBV infection takes place at a younger age in low SES regions or less developed countries, while it is often delayed to adolescence for primary EBV infection in high SES areas [2,5–7]. Several studies have also demonstrated that EBV seroprevalence differs by sex [6–8]. Knowledge of these differences is important for the development of targeted interventions to reduce the morbidity of EBV-related diseases [7,9].

Vaccination could prevent EBV infection and has the potential to reduce the incidence of EBV-related diseases [2]. Currently there is no licensed EBV vaccine, however some candidates show promise in clinical trials, such as the gp350-based EBV vaccine [1]. This first-generation vaccine protected against IM but did not prevent infections [1].

Recent developments have led to EBV vaccine formulations that induce a stronger immune response than that observed with the original gp-350 vaccine, target a broader range of viral glycoproteins, and use improved methods of delivery [10,11]. Results from preclinical trials of these vaccines have been promising and led to the initiation of early phase human trials that will hopefully result in vaccines with expanded protection against EBV infection and its associated diseases [10].

Despite the importance of EBV as a cause of infectious mononucleosis, several cancer types, and autoimmune diseases, information on the epidemiology of EBV infection in Costa Rica is limited. To our knowledge, no population-based study has previously described age-specific EBV seroprevalence in Costa Rican population.

We conducted this study to evaluate the age-specific prevalence of EBV in Costa Rica. Our objective was to understand patterns of EBV acquisition to contribute to a better understanding of the epidemiology of EBV in Costa Rica, which, in turn, may inform strategies for addressing EBV infection in the future.

## Materials and methods

### Parent cohort

The present study is a cross-sectional analysis of baseline data in the RESPIRA cohort. The RESPIRA (Evaluation of the immune response to SARS- CoV- 2 in Costa Rica) study is a population-based cohort study conducted in Costa Rica to investigate the immune response to SARS-CoV-2 infection. Detailed study methods have been described in a previous publication [12]. In summary, RESPIRA included an age-stratified sample of 999 Polymerase Chain Reaction (PCR)-confirmed COVID-19 cases selected from the national COVID-19 registry and 1,999 population controls matched to cases on age, sex, and geographic residence. Cases and controls were recruited from regions of the country covering approximately 70% of its population. Participants were enrolled between November 2020 and October 2021. The participation rate at enrollment was 61% among COVID-19 cases and 73% among population controls in the RESPIRA cohort [12].

At enrollment, all participants completed a detailed questionnaire that obtained information on sociodemographic factors, including age, sex and urbanicity. Blood was collected, from which serum was obtained and stored in a frozen state. The study was approved by the Central Social Security (CCSS) Institutional Review Board (IRB) and all participants or their legal representatives signed informed consent or assent (for children 12–17 years old) in the presence of a witness, as mandated by Costa Rican law.

The RESPIRA study’s broad representation of the Costa Rican population makes it a suitable parent cohort for investigating Epstein-Barr virus prevalence. Study participants reflected the study area with respect to sex and geographic distribution S1 Table.

### Selection of samples for EBV antibody testing

All enrollment samples from participants recruited into the cohort were selected for EBV antibody testing using the Diasorin LIAISON XL VCA IgG assay. In total, we selected and tested 2,990 individuals, including 554 children and adolescents <20, 1,740 adults 20–59, and 696 adults 60 + .

### Selection of samples for quality control

To facilitate direct comparison of our findings with those presented in a previous survey of EBV seropositivity conducted within a population-based cohort in the United States, the National Health and Nutrition Examination Survey (NHANES) cohort [2], a subset of 278 samples was selected for EBV antibody testing using the Diamedix EBV VCA IgG Enzyme Immunoassay (EIA) used in the NHANES cohort. This subset was selected to have a broad age representation, while oversampling children and adolescents, given the expectation that EBV acquisition typically occurs before adulthood and that seropositivity among adults was likely to be close to 100%.

To document the reproducibility of assay results, we additionally selected blinded duplicates for retesting by each of the assays; 150 for the Diasorin assay and 25 for the Diamedix assay.

### Testing methods

The Diasorin LIAISON XL VCA IgG assay (Diasorin, Saluggia, Italy) was used for EBV antibody testing at the main pediatric hospital in Costa Rica (Hospital Nacional de Niños). Testing was performed per manufacturer instructions. In brief, the Diasorin Liaison XL VCA IgG assay is an indirect chemiluminescent immunoassay (CLIA) that utilizes magnetic particles coated with EBV VCA p18 synthetic peptides and mouse monocloncal antibodies to human IgG to detect presence of EBV VCA IgG antibodies in 20 µL serum. Presence of antigen-bound magnetic particles following washing is detected by a chemiluminescent signal reported out as relative light units (RLUs). Samples with RLU of 20 + were considered positive; those with RLU < 20 were considered negative. Among the 150 samples tested in duplicate, we observed a coefficient of variation (%CV) of 10%, a Pearson correlation of 0.98, and an interclass correlation coefficient (ICC) of 0.98. When the dichotomous outcome was evaluated, the overall percent agreement was 99%, the agreement among negatives was 96%, and the Kappa was 0.97. Only one inconsistent result was observed where one value was below the positivity threshold (0 RLU) and the other slightly above it (23.7 RLU).

The Diamedix EBV VCA IgG EIA assay (Diamedix Corporation, Miami, USA) was employed at the University of Minnesota. This enzyme immunoassay detects IgG antibodies to the EBV viral capsid antigen (VCA). The Diamedix EBV VCA IgG EIA assay was performed using 20 µL of serum, which was pre-diluted at a ratio of 1:21. The diluted samples were placed in labeled wells and underwent incubation, followed by washing. After washing, a conjugate was added, and the samples were incubated again. The wells were washed a second time, after which a substrate was added and incubated. Finally, a stop solution was applied to halt the reaction. The results were measured by reading the plates at an absorbance of 450 nm, with a reference wavelength of 630 nm. EIA indices were manually calculated based on the kit’s formulas. Samples were classified as positive if the EIA index was 1.10 + , negative if <0.90, and borderline if between 0.90 and 1.09. For purposes of this analysis, samples (N = 4) with EIA levels in the borderline range were classified as positive. Among the 25 samples tested in duplicate, we observed a %CV of 14%, a Pearson correlation of 0.87 and an ICC of 0.86. When the dichotomous outcome was evaluated, the overall percent agreement was 96%, the agreement among negatives was 80%, and the Kappa was 0.86. The single inconsistency noted between duplicate results were close to the positivity threshold (1.15 and 0.81).

For the 278 samples tested by both assays, we observed a Pearson correlation between assays of 0.86 when comparing log-transformed results used in the analyses. For the dichomomous readings, we observed an overall percent agreement of 96%, an agreement among negatives of 73%, and a Kappa of 0.82.

### Statistical analysis

The primary analysis focused on determining the seroprevalence of Epstein-Barr Virus (EBV) by age. Additionally, we calculated the geometric mean levels (GML) of EBV VCA IgG antibodies within the seropositive group to represent antibody levels among those previously exposed to EBV. Participants were divided into several age categories (0−4, 5−9, 10−14, 15−19, 20−29, 30−39, 40−49, 50−59, 60−69, 70−79, and 80 + years). Sensitivity analyses were performed stratified by case-control status in the *RESPIRA* study (COVID-19 cases versus population controls) to assess whether the overall patterns observed in the primary analyses differed between these groups.

In addition, we assessed age-specific sero-prevalence for consistency and GMLs among seropositives by urbanicity and sex. Degree of urbanicity was defined at the district level based on the participant’s residential address at enrollment and classified as follows; 1) rural/mixed areas, 2) urban areas outside the San José (capital city) greater metropolitan area (GAM), and 3) urban areas within the GAM using previously reported criteria [13]. To avoid small numbers in these stratified analyses, we evaluated children/adolescents in three age groups: 0- < 5, 5- < 10, and 10- < 20. Additionally, as seropositivity was invariably above 95% among adults 20 and older, we combined adults into two broad groups: 20- < 65 and 65 + .

## Results

A total of 2,990 individuals were included in the present analysis. These individuals had a median age of 41 years and 47% were men. 57% were from urban/metropolitan areas, 16% from urban/non-metropolitan areas, and 27% from rural/mixed areas.

Fig 1 and S2 Table presents the percentage of EBV seropositivity (orange dots; Y1-axis) and the GML of EBV VCA antibodies among EBV seropositive individuals (blue bars; Y2-axis) by age. Seropositivity increased with age (p < 0.01), rising from 20% [95% CI: 8−39] in children <2 years of age to 100% [95% CI: 93−100] in individuals aged 80 + . Seropositivity reached 80% (95% Confidence Interval (CI): 70−88) among children aged 6– < 10 and exceeded 90% by age 20. The GML among seropositives also increased with age (p < 0.01), with values stable in childhood and adolescence at around 200 median fluorescense intensity (MFI) and rising continuously during adult life to above 400 among those 70 + . Results were comparable in sensitivity analysis stratified by case-control status S3 Table 3, supporting our decision to combine COVID-19 cases and controls for the primary analysis.

**Fig 1:**
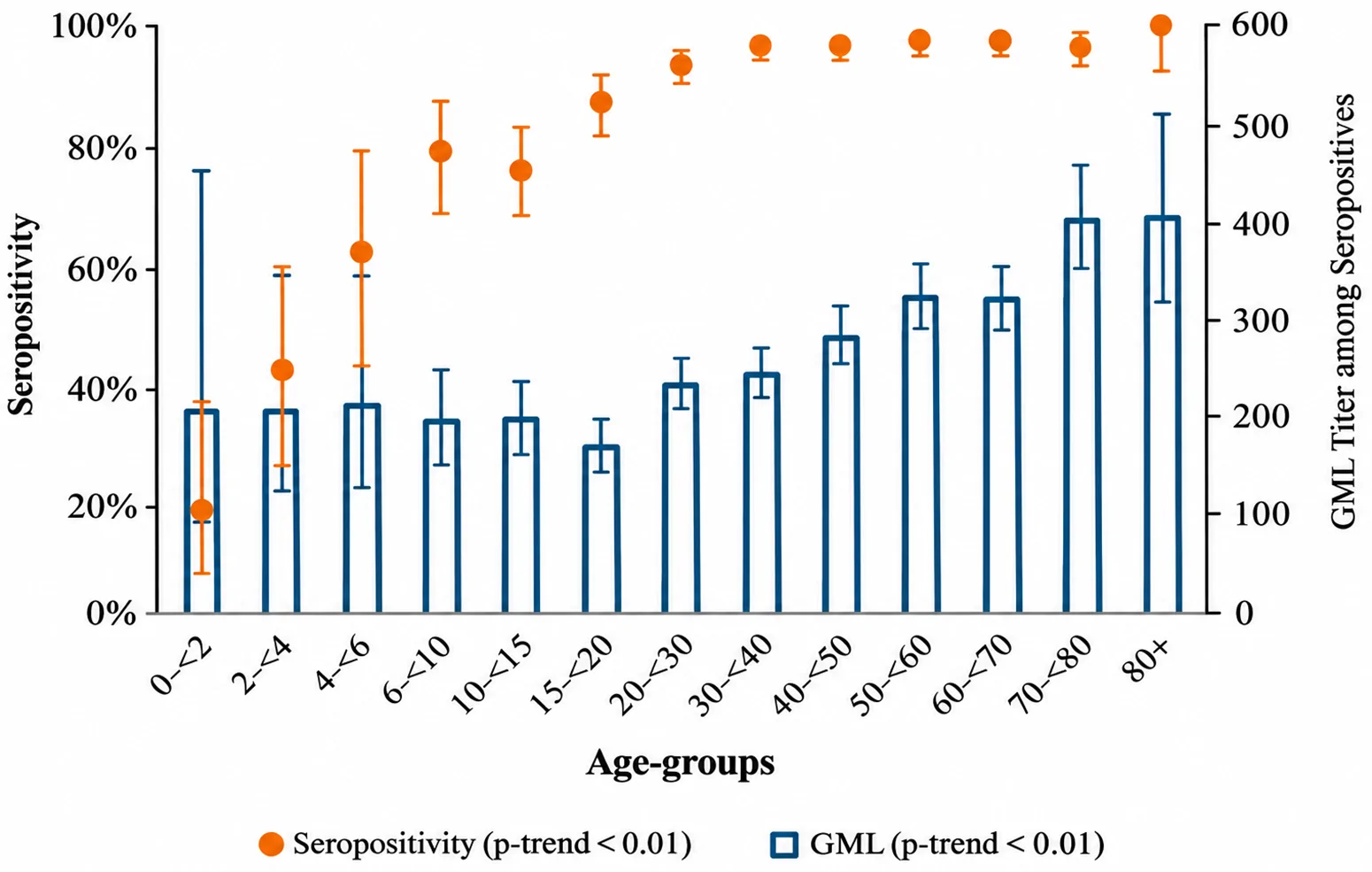
EBV seropositivity and Geometric Mean Level (GML) among seropositives, by age. Error bars represent 95% confidence intervals.

Age-specific seroprevalence and GML among seropositives stratified by sex and urbanicity are presented in Table 1. Overall, we observed similar patterns among men and women, with seropositivity and GMLs among seropositives increasing with age among both men and women. However, a few differences were noted. First, we observed that seropositivity among children 0- < 5 was suggestively higher among boys than girls (45% [95% CI: 32–59] for boys vs 23% [95% CI: 10–41] for girls; p-value 0.046). In adolescence, this pattern was reversed, with women having higher seropositivity than men (88% for women [95% CI: 83–92] vs 77% for men [95% CI: 70–83]; p-value = < 0.004.) Among adults, both men and women reached seropositivity rates nearing 100%. Second, we noted that among seropositive individuals, GMLs were consistently higher among women compared with men at all ages, with levels at ages 20–64 being significantly higher in women compared to men (GML = 326 MFI for women vs 260 MFI for men; p-value <0.01).

Observed patterns were similar by urbanicity, with seropositivity and GMLs among seropositives increasing with age in all three groups. A younger age at seroconversion, however, was suggested in rural/mixed areas, where seropositivity among children 0- < 5 years was 50% (95% CI: 16–84) compared to 32% (95% CI: 15–54) in urban/not GAM areas and 38% (95% CI: 25–52) among urban/GAM areas (p-value = 0.05). This pattern of earlier age at seroconversion in less urban areas was reinforced by the lower seropositivity 75% (95% CI: 62–86) observed in individuals 5- < 10 years from urban/GAM areas compared with those from other regions of Costa Rica (seropositivity among those 5- < 10 years = 88% (95% CI: 69–97) in rural/mixed areas and 87% (95% CI: 60–98) in urban/not GAM areas; p-value = 0.05).

**Table 1:** Age-specific EBV seropositivity and Geometric Mean Level (GML) by sex and urbanicity.

A. BY SEX											
	N		% Seropositive			GML in Seropositives					
Age Group	Men	Women	Men	Women	p-value	Men	Women	p-value			
0-<5yrs	55	31	45% [32-59]	23% [10-41]	0.046	216 [151-307]	300 [117-770]	0.365			
5-<10yrs	49	48	78% [63-88]	83% [70-93]	0.484	186 [132-262]	239 [180-317]	0.280			
10-<20yrs	167	204	77% [70-83]	88% [83-92]	0.004	176 [147-211]	215 [187-247]	0.081			
20-<65yrs	894	1073	97% [96-98]	96% [95-97]	0.588	260 [243-278]	326 [307-345]	p<0.001			
65+ yrs	252	217	97% [94-99]	99% [96-100]	0.300	356 [315-401]	401 [357-450]	0.124			
**B. BY URBANICITY**											
	**N**			**% Seropositive**				**GML in Seropositives**			
**Age Group**	**Rural/Mixed**	**Urban/Not GAM**	**Urban/GAM**	**Rural/Mixed**	**Urban/Not GAM**	**Urban/GAM**	**p-value**	**Rural/Mixed**	**Urban/Not GAM**	**Urban/GAM**	**p-value**
0-<5yrs	8	25	53	50% [16-84]	32% [15-54]	38% [25-52]	0.666	349 [77-1580]	307 [175-536]	191 [123-296]	0.054
5-<10yrs	25	15	57	88% [69-97]	87% [60-98]	75% [62-86]	0.412	225 [145-350]	222 [138-357]	202 [148-276]	0.965
10-<20yrs	98	63	210	88% [80-94]	89% [78-95]	80% [73-85]	0.127	202 [165-248]	216 [166-281]	190 [163-222]	0.735
20-<65yrs	584	281	1102	96% [94-97]	97% [94-99]	97% [95-98]	0.848	293 [271-317]	267 [236-303]	301 [284-320]	0.155
65+ yrs	102	80	287	98% [93-100]	99% [93-100]	98% [95-99]	0.704	396 [333-471]	331 [261-418]	383 [344-425]	0.506

## Discussion

This study defines age-related patterns of EBV seroprevalence and antibody levels in Costa Rica. Seropositivity increased with age, reaching 80% by ages 5– < 10 and exceeded 90% among adults. GML of EBV antibodies among seropositive individuals rose with age, continuing to increase during adult life and peaking in older adults. Some sex-specific differences were observed: boys had higher seropositivity in early childhood, while seropositive girls and women exhibited consistently higher antibody levels across all age groups. There was also some evidence for earliest acquisition of EBV infection in rural areas, followed by urban areas outside of the capital city of San Jose, representing lower socio-economic status areas in the San Jose metropolitan area, and later acquisition in the urban areas of the capital city of San Jose.

When comparing Costa Rica to other countries such as the United States, some differences were noted. In Costa Rica, acquisition occurs earlier than in the Unites States, with 76% of children already infected by the age of 10, compared to approximately 50% among children of a similar age in the United States [2]. Nonetheless, in both countries, positivity rates reach/exceed 90% among adults [2]. These observations suggest that Costa Rican children are exposed to EBV earlier in life, likely due to differences in living/environmental conditions. Both countries show that antibody levels rise with age and that women tend to have higher levels than men [2].

The observed age-specific seroprevalence of EBV has implications for future interventions aimed at controlling EBV and its associated conditions. For instance, interventions to prevent initial infection (e.g., prophylactic vaccination) should target young children since the seroprevalence exceeds 50% by ages 4–6 years and reaches 80% by ages 6–10 years. In contrast, interventions (e.g., therapeutic vaccines or screening/early detection strategies) developed to manage EBV post-infection to minimize clinical sequelae including EBV-driven malignancies or auto-immunity could be directed towards adolescents or young adults.

In addition to seroprevalence, antibody levels among seropositives also increased with age in our study. In fact, seropositive individuals aged 70 + years had average EBV antibody levels that were nearly double those among young children and adolescents. This finding is consistent with our understanding of EBV infection, which establishes lifelong, latent infection in B-cells that periodically reactivate throughout life, providing repeated opportunities for viral exposure and boosting of antibody levels across a lifetime [14,15].

Sex differences in immune response have been reported [14,15]. The higher antibody levels observed in our and previous studies among females compared with males might partially explain the fact that females have higher rates of auto-immune conditions than males [14–17].

Patterns of rural/urban EBV seroprevalence observed in our study in Costa Rica are consistent with findings reported in other populations, including studies from Taiwan, the United Kingdom, and the Netherlands [5–7]. In these populations, EBV infection tends to be acquired at younger ages in rural areas with lower socio-economic levels and increases in more urban and affluent areas. Whether these differences will affect the impact of future interventions aimed at controlling EBV infection and its associated clinical conditions remains to be seen.

Finally, some strengths and limitations of our study warrant mention. The RESPIRA cohort is a large, population-based sample that offers a broad geographic and demographic representation of Costa Rica. The use of a validated, reproducible testing method, along with re-testing a subset of samples using a second, well-established assay, is also a strength. Limitations of our work include its cross-sectional design, which precluded us from evaluating infection incidence at the individual level, and may have introduced cohort effects. Changes in living conditions and other social factors over time could have influenced the age at EBV acquisition across generations. In addition, because the study was designed to describe age-specific patterns of EBV seroprevalence in the population, the analyses were primarily descriptive and were not intended to identify causal determinants of infection. The modest sample size for some of our subgroup analyses, especially for analyses of age-specific EBV seroprevalence by sex and urbanicity, may have limited the precision of some estimates.

## Supporting information

S1 Table Geographic and sex distribution of the RESPIRA study. (DOCX)

S2 Table Percent EBV seropositivity and Geometric Mean Level (GML) among seropositives, by age. (DOCX)

S3 Table Age-specific EBV seropositivity and Geometric Mean Level (GML) by COVID-19 status. (DOCX)
