# Supplementary material for: Age-specific Epstein-Barr virus seroprevalence in Costa Rica
Source: PLoS One. 2026 Nov 10;21(11):e0362125. doi: 10.1371/journal.pone.0362125 (PMC13750545; doi:10.1371/journal.pone.0362125)
Supplement: Supplementary file 1 [file pone.0362125.s001.docx]

**Supplemental Table 1: Geographic and Sex Distribution of the RESPIRA Study.**

|  | Study Sample  N (%) | Study Area  N (in thousand) (%) |
| --- | --- | --- |
| **Total** | 2990 | 3608 (100) |
|  |  |  |
| **Sex** |  |  |
| Men | 1417 (47) | 1807 (50) |
| Women | 1573 (53) | 1801 (50) |
|  |  |  |
| **Age** |  |  |
| 0-19 | 554 (19) | 986 (27) |
| 20-39 | 872 (29) | 1211 (34) |
| 40-59 | 868 (29) | 885 (25) |
| 60+ | 696 (23) | 526 (15) |
|  |  |  |
| **Area** |  |  |
| Rural/Mixed | 817 (27) | 878 (24) |
| Urban (No-GAM) | 464 (16) | 342 (9) |
| Urban (GAM) | 1709 (57) | 2388 (66) |
