# Supplementary material for: Age-specific Epstein-Barr virus seroprevalence in Costa Rica
Source: PLoS One. 2026 Nov 10;21(11):e0362125. doi: 10.1371/journal.pone.0362125 (PMC13750545; doi:10.1371/journal.pone.0362125)
Supplement: Supplementary file 2 [file pone.0362125.s002.docx]

**Supplemental Table 2: Percent EBV Seropositivity and Geometric Mean Level (GML) Among Seropositives, by Age**

| **Age Group**  Trend (p-value) | **N** | **%Seropositive**  **p<0.01** | **95% CI** | **N** | **GML Among Seropositives**  **p<0.01** | **95% CI** |
| --- | --- | --- | --- | --- | --- | --- |
| **0<2** | 30 | 20% | [8 – 39] | 6 | 224 | [109 – 459] |
| **2<4** | 36 | 44% | [28 – 62] | 16 | 223 | [139 – 359] |
| **4<6** | 33 | 64% | [45 – 80] | 21 | 227 | [143 – 360] |
| **6<10** | 84 | 80% | [70 – 88] | 67 | 212 | [168 – 269] |
| **10<15** | 145 | 77% | [70 – 84] | 112 | 215 | [181 – 256] |
| **15<20** | 226 | 87% | [82 – 91] | 197 | 189 | [164 – 218] |
| **20<30** | 370 | 94% | [91 – 96] | 347 | 252 | [227 – 279] |
| **30<40** | 502 | 97% | [95 – 98] | 485 | 265 | [242 – 290] |
| **40<50** | 446 | 97% | [95 – 98] | 431 | 302 | [275 – 332] |
| **50<60** | 422 | 98% | [96 – 99] | 413 | 339 | [309 – 373] |
| **60<70** | 440 | 98% | [96 – 99] | 432 | 337 | [307 – 369] |
| **70<80** | 202 | 97% | [93 – 99] | 195 | 416 | [369 – 469] |
| **80+** | 54 | 100% | [93 – 100] | 54 | 417 | [335 – 519] |
