# Supplementary material for: Age-specific Epstein-Barr virus seroprevalence in Costa Rica
Source: PLoS One. 2026 Nov 10;21(11):e0362125. doi: 10.1371/journal.pone.0362125 (PMC13750545; doi:10.1371/journal.pone.0362125)
Supplement: Supplementary file 3 [file pone.0362125.s003.docx]

**Supplemental Table 3: Age-Specific EBV Seropositivity and Geometric Mean Level (GML) by COVID-19 Status**

|  | COVID-19 Cases | | | | Population Controls | | | | p-value* | | |
| --- | --- | --- | --- | --- | --- | --- | --- | --- | --- | --- | --- |
| Age Group | N | % Seropositive (95% CI) | GML in Seropositives (95% CI) | N | | % Seropositive (95% CI) | GML in Seropositives (95% CI) | For % Seropositive | | For GML in Seropositives |  |
| 0-<2yrs | 14 | 14% [2 – 43] | 245 [11 – 5468] | 16 | | 25% [7 – 52] | 214 [55 – 835] | 0.469 | | 0.676 |  |
| 2-<4yrs | 12 | 42% [15 – 72] | 206 [58 – 730] | 24 | | 46% [26 – 67] | 231 [128 – 418] | 0.648 | | 0.553 |  |
| 4-<6yrs | 9 | 78% [40 – 97] | 177 [62 – 509] | 24 | | 58% [37 – 78] | 258 [148 – 449] | 0.494 | | 0.557 |  |
| 6-<10yrs | 28 | 86% [67 – 96] | 172 [117 – 253] | 56 | | 77% [64 – 87] | 239 [177 – 323] | 0.312 | | 0.238 |  |
| 10-<15yrs | 26 | 73% [52 – 88] | 193 [123 – 303] | 119 | | 78% [70 – 85] | 220 [181 – 266] | 0.520 | | 0.476 |  |
| 15-<20yrs | 96 | 92% [84 – 96] | 207 [166 – 258] | 130 | | 84% [76 – 90] | 175 [145 – 212] | 0.103 | | 0.217 |  |
| 20-<30yrs | 122 | 96% [91 – 99] | 247 [206 – 296] | 248 | | 93% [89 – 96] | 255 [224 – 289] | 0.253 | | 0.639 |  |
| 30-<40yrs | 167 | 96% [92 – 99] | 289 [249 – 336] | 335 | | 97% [94 – 98] | 253 [226 – 283] | 0.931 | | 0.104 |  |
| 40-<50yrs | 151 | 96% [92 – 99] | 307 [261 – 362] | 295 | | 97% [94 – 99] | 300 [267 – 337] | 0.610 | | 0.715 |  |
| 50-<60yrs | 140 | 96% [92 – 99] | 295 [249 – 350] | 282 | | 99% [96 – 100] | 363 [324 – 407] | 0.190 | | 0.062 |  |
| 60-<70yrs | 149 | 97% [92 – 99] | 299 [253 – 353] | 291 | | 99% [97 – 100] | 357 [319 – 399] | 0.092 | | 0.074 |  |
| 70-<80yrs | 62 | 95% [87 – 99] | 441 [359 – 541] | 140 | | 97% [93 – 99] | 406 [349 – 471] | 0.534 | | 0.442 |  |
| 80+ yrs | 21 | 100% [84 – 100] | 409 [271 – 618] | 33 | | 100% [89 – 100] | 422 [325 – 549] | 1.000 | | 1.000 |  |

* p-value between groups
